# miR‐19b controls cardiac fibroblast proliferation and migration

**DOI:** 10.1111/jcmm.12858

**Published:** 2016-04-06

**Authors:** Chongjun Zhong, Kun Wang, Ying Liu, Dongchao Lv, Bo Zheng, Qiulian Zhou, Qi Sun, Ping Chen, Shengguang Ding, Yiming Xu, Haitao Huang

**Affiliations:** ^1^ Department of Thoracic and Cardiovascular SurgeryThe Second Affiliated Hospital of NanTong UniversityNantongChina; ^2^ Department of CardiologyShanghai Putuo District Central HospitalShanghai University of Traditional Chinese MedicineShanghaiChina; ^3^ School of Life ScienceShanghai UniversityShanghaiChina; ^4^ Shanghai Institute of Biological Products Co., LtdShanghaiChina

**Keywords:** cardiac fibrosis, miR‐19b, Pten

## Abstract

Cardiac fibrosis is a fundamental constituent of a variety of cardiac dysfunction, making it a leading cause of death worldwide. However, no effective treatment for cardiac fibrosis is available. Therefore, novel therapeutics for cardiac fibrosis are highly needed. Recently, miR‐19b has been found to be able to protect hydrogen peroxide (H_2_O_2_)‐induced apoptosis and improve cell survival in H9C2 cardiomyocytes, while down‐regulation of miR‐19b had opposite effects, indicating that increasing miR‐19b may be a new therapeutic strategy for attenuating cellular apoptosis during myocardial ischaemia–reperfusion injury. However, considering the fact that microRNAs might exert a cell‐specific role, it is highly interesting to determine the role of miR‐19b in cardiac fibroblasts. Here, we found that miR‐19b was able to promote cardiac fibroblast proliferation and migration. However, miR‐19b mimics and inhibitors did not modulate the expression level of collagen I. Pten was identified as a target gene of miR‐19b, which was responsible for the effect of miR‐19b in controlling cardiac fibroblast proliferation and migration. Our data suggest that the role of miR‐19b is cell specific, and systemic miR‐19b targeting in cardiac remodelling might be problematic. Therefore, it is highly needed and also urgent to investigate the role of miR‐19b in cardiac remodelling *in vivo*.

## Introduction

Cardiac fibrosis represents a fundamental constituent of many cardiac dysfunction, including dilated, ischaemic, and hypertrophic cardiomyopathies, myocardial infarction and heart failure 1, 2. In addition, cardiac fibrosis can also be a primary disease independent of pre‐existing cardiomyocyte injury 3, 4, 5, 6. Cardiac fibrosis has been constituted as a major cause of death worldwide due to the fact that it can increase cardiac stiffness, impair cardiomyocyte contractility and disrupt electrical coupling 7. However, currently there is no effective treatment for cardiac fibrosis in general 1, 4. Therefore, novel therapeutics for cardiac fibrosis are highly needed 3.

MicroRNAs (miRNAs, miRs) are endogenous, small 19–25 nucleotide non‐protein‐coding single‐stranded RNAs that inhibit target gene expressions by repressing translation or inducing degradation of its target mRNA or both 8, 9, 10. At present, about 2000 miRNAs have been reported in human 11, 12. Considering the fact that a single miRNA can regulate multiple mRNA targets while a single mRNA target can be regulated by several miRNAs, it is generally accepted that nearly 60% of human mRNAs are regulated by miRNAs 13, 14, 15. As important regulators of almost all aspects of cardiac biology, it is not surprising that miRNAs regulate cardiomyocyte hypertrophy, apoptosis, proliferation and metabolism 16, 17, 18. Dysregulation of miRNAs has already been linked to a large number of cardiovascular diseases including cardiac fibrosis 5, 19. Let‐7, miR‐21, miR‐22, miR‐34a, miR‐208a, miR‐377 and miR‐652 have been reported to promote the genesis of cardiac fibrosis, while miR‐1, miR‐15 family, miR‐24, miR‐26a, miR‐29b, miR‐101, miR‐122, miR‐133, miR‐145, miR‐378 and miR‐489 attenuate the fibrotic response of heart 6, 20, 21. These reports suggest miRNA as powerful regulators of cardiac fibrosis 6, 20, 21.

The miR‐17/92 cluster is a best‐explored miRNA cluster and its dysregulation has been related to many diseases including cardiovascular diseases 22, 23, 24, 25. Among the miR‐17/92 cluster, miR‐19b is a key component 22, 25, 26. Recently, it has been reported that miR‐19b was able to protect hydrogen peroxide (H_2_O_2_)‐induced apoptosis and improve cell survival in H9C2 cardiomyocytes, while down‐regulation of miR‐19b had inverse effects, indicating that increasing miR‐19b may be a new therapeutic strategy for attenuating cellular apoptosis during myocardial ischaemia–reperfusion injury 27. Considering the fact that miRNAs might exert a cell‐specific role 19, it is highly interesting to determine the role of miR‐19b in cardiac fibroblasts.

Cardiac fibroblasts are a major type of cells responsible for cardiac fibrosis 20. The proliferation, migration and activation of cardiac fibroblasts play an important role in the genesis of cardiac fibrosis 6, 20, 28, 29. Activated cardiac fibroblasts are featured with increased synthesis of protein, including collagen I, the predominant extracellular matrix protein 20. In this study, we found that miR‐19b was able to promote cardiac fibroblast proliferation and migration. Phosphatase and tensin homologue (Pten) was identified as a target gene of miR‐19b. Our data highly suggested that it was urgent to determine the role of miR‐19b in cardiac remodelling *in vivo*. Moreover, as the role of miR‐19b is cell specific, systemic miR‐19b targeting in cardiac remodelling might be problematic.

## Materials and methods

### Cardiac fibroblast isolation and culture

Neonatal rat cardiac fibroblasts (NRCFs) were isolated from the heart of 0–3 day old Sprague Dawley SD rats. Ventricle tissues were minced and digested in 0.06% collagenase (Gibco, San Diego, CA, USA) and 0.04% pancreatin (Sigma‐Aldrich, St. Louis, MO, USA) containing solution at 37°C. All cells were pre‐plated for 1 hr so that NRCFs could rapidly adhere to the plate. After that, cardiomyocytes were removed and NRCFs were cultured in Dulbecco's modified Eagle's medium (DMEM; Corning, NY, USA) containing 10% Fetal Bovine Serum (BI, Beit‐Haemek, Israel), 100 mg/ml streptomycin and 100 U/ml penicillin. All cells were maintained in a 37°C incubator containing 5% CO_2_. These NRCFs were defined as passage 0 (P0). NRCFs were passaged once every 4 days, and NRCFs at passage 2 (P2) were used in the following studies.

### Cell transfection

miR‐19b mimics, negative‐mimics, miR‐19b inhibitors and negative‐inhibitors were all purchased from RiboBio (Guangzhou, China). The small interfering RNAs (siRNAs) for Pten and the negative controls were also obtained from RiboBio (Guangzhou, China). NRCFs at a density of 0.2 million cells/ml were seeded in 96‐well, 24‐well, 12‐well and 6‐well plates for different studies. After starving in 1% of FBS for at least 8 hrs before transfection, miR‐19b mimics (50 nM), miR‐19b inhibitors (100 nM), Pten siRNAs (100 nM) and their negative controls were transfected into NRCF using Lipofectamine 2000 (Invitrogen, Carlsbad, CA, USA). After 48 hrs, different experiments were performed.

### Cell proliferation assay

Forty‐eight hours after miR‐19b mimics, miR‐19b inhibitors, and their negative‐controls transfection, cell proliferation was analysed by Cell Counting Kit (CCK‐8, Dojindo, Japan) and 5‐ethynyl‐2′‐deoxyuridine (EdU) Kit (RiboBio, Guangzhou, China). CCK‐8 was added 1 hr before the terminal time of the experiment and was incubated in the 37°C incubator. The data were analysed by a Microplate Absorbance Reader (Bio‐rad, Richmond, CA, USA) under the absorbance of 450 nm. During EdU assays, NRCFs were incubated with 10 μM EdU for 24 hrs before tested.

### Cell migration assay

NRCFs at P2 were plated in the 12‐well plate. After forming the monolayer, a wound was made by a line each well with a 200‐μl pipette tip. Cell migration was determined by the width of the initial scratched area at 0 hr and the width of the terminal scratched area at 48 hrs.

### Quantitative real‐time reverse transcriptase‐polymerase chain reactions (qRT‐PCRs)

Total RNA was extracted from NRCFs using the Trizol Reagent (Invitrogen). For mRNA detection, 400 ng of total RNA was used to the reverse transcription reaction *via* Bio‐Rad iScript^™^ cDNA Synthesis Kit (Bio‐Rad). The RT product was subjected to 40 cycles of quantitative PCR with Takara SYBR Premix Ex Taq^™^ (Tli RNaseH Plus, TaKara, Dalian, Liaoning Province, China) in a CFX96TM Real‐Time PCR Detection System (Bio‐Rad). 18S was used to normalize Pten gene. The sequences of Pten primer: forward CAATGTTCAGTGGCGAACTT (5′‐3′) and reverse GGCAATGGCTGAGG GAACT (5′‐3′). The sequences of 18S primer: forward ATTCGAACGTCTGCCC TATCAA (5′‐3′) and reverse CGGGAGTGGGTAATTTGCG (5′‐3′).

The Bulge‐Loop^™^ miRNA qRT‐PCR Primer Set (Ribobio, Guangzhou, China) was used for reverse transcription reaction *via* the Bio‐Rad iScript^™^ cDNA Synthesis Kit (Bio‐Rad). The Takara SYBR Premix Ex Taq^™^ (Tli RNaseH Plus) was used to determine the expression level of miR‐19b by qRT‐PCRs in the CFX96 Real‐time PCR Detection System. 5S was used to normalize the expression of miR‐19b. Relative expression levels for each mRNA and miRNA expression were calculated by the 2^−▵▵CT^ method.

### Western blotting

NRCFs were lysed in RIPA buffers (KeyGene, Nanjing, Jiangsu Province, China) containing 1% phenylmethanesulfonyl fluoride (PMSF). Total proteins were quantified using the BCA protein assay reagent kit (KeyGene, China). Proteins were separated in 10% SDS‐PAGE gels *via* electrophoresis and transferred onto PVDF membranes. Standard western blot analysis used PTEN (1:1000 dilution; Abcam; ab133532) and Col‐1 (1:1000 dilution; Bioworld; BS1530) as primary antibodies incubated overnight in 4°C. The β‐actin antibody (1:10000 dilution; Abclonal; AC004) was used as the internal reference. After the appropriate HRP Goat Anti‐Mouse IgG (1:10000 dilution; Abclonal; AS003) was incubated for 2 hrs at room temperature, the ECL System (Bio‐rad) was used to visualize the signal *via* the ChemiDoc XRS Plus luminescent image analyser (Bio‐Rad).

### Statistical analysis

All data were presented as mean ± SEM, and an independent‐sample *t*‐test was used to calculate all data *via* SPSS version 19. *P* < 0.05 was settled as the limit of statistical significance.

## Results

### miR‐19b promotes cardiac fibroblast proliferation

**Figure 1:**
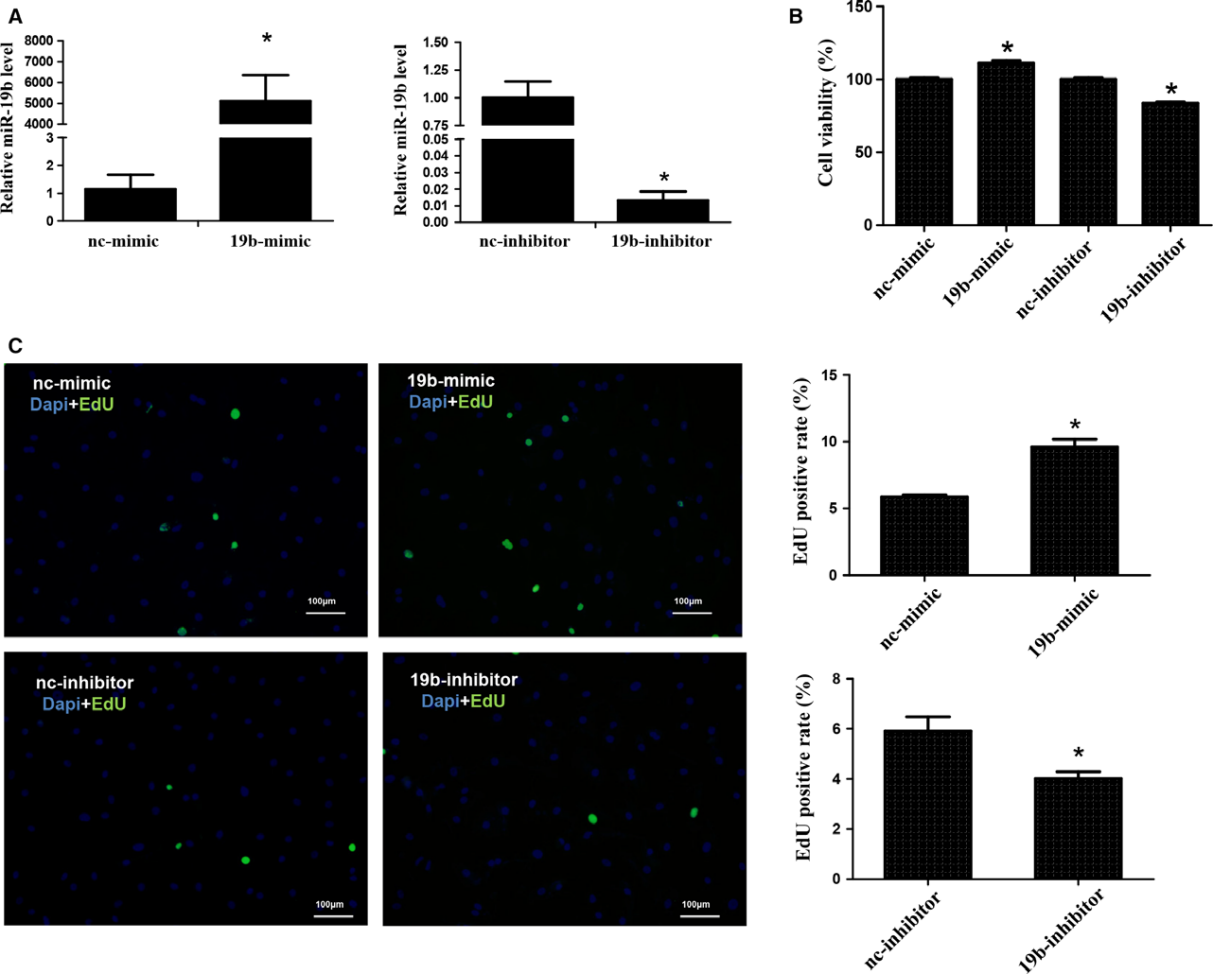
miR‐19b promotes cardiac fibroblast proliferation. Quantitative real‐time reverse transcriptase‐polymerase chain reactions indicated that miR‐19b mimics increased while miR‐19b inhibitors decreased miR‐19b expression in cardiac fibroblasts (**A**). miR‐19b promoted cardiac fibroblast proliferation as evidenced by CCK‐8 (**B**) and EdU staining assays (**C**). Scale bar, 100 μm. *P < 0.05.

To investigate the role of miR‐19b in cardiac fibroblasts, cardiac fibroblasts were transfected with miR‐19b mimics or miR‐19b inhibitors to overexpress or knock‐down miR‐19b, respectively. Forty‐eight hours after transfection, qRT‐PCRs were used to determine the expression level of miR‐19b. We confirmed that transfection with 50 nM miR‐19b mimics increased miR‐19b expression, whereas transfection with 100 nM miR‐19b inhibitors decreased that (Fig. 1A). miR‐19b mimics were found to be able to promote cardiac fibroblast proliferation as evidenced by both CCK‐8 and EdU assays (Fig. 1B and C), while miR‐19b inhibitors had opposite effects (Fig. 1B and C). Collectively, our data suggest that miR‐19b was both sufficient and required for cardiac fibroblast proliferation.

### miR‐19b enhances cardiac fibroblast migration

**Figure 2:**
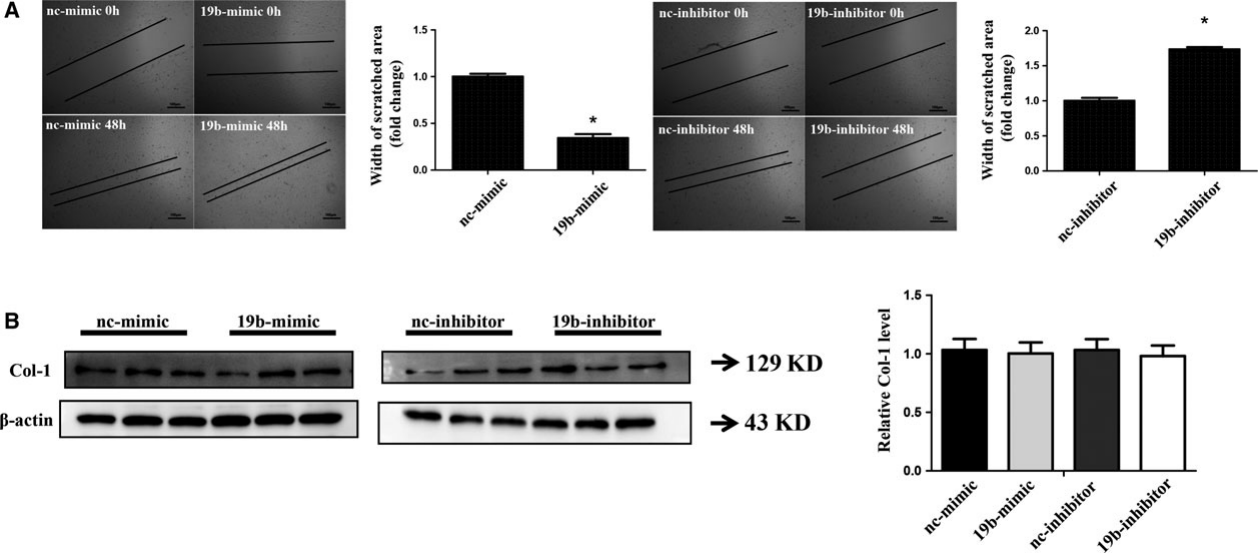
miR‐19b enhances cardiac fibroblast migration but not changes collagen I. (**A**) miR‐19b mimics promoted cardiac fibroblast migration (as evidenced by the decreased unhealing distance) while miR‐19b inhibitors decreased that (as evidenced by the increased unhealing distance). (**B**) miR‐19b mimics and inhibitors did not modulate the expression level of collagen I. Scale bar, 100 μm. *P < 0.05.

The regulatory effect of miR‐19b on migration was determined based on unhealing distance. The smaller the unhealing distance, the greater the migration capacity. It was found that miR‐19b mimics significantly decreased the unhealing distance of cardiac fibroblasts, while miR‐19b inhibitors increased that (Fig. 2A), indicating that miR‐19b was a positive regulator of cardiac fibroblast migration.

As collagen synthesis is also a major function of cardiac fibroblasts 21, 30, we determined the effect of miR‐19b mimics and inhibitors in regulating collagen I expression. However, miR‐19b mimics and inhibitors did not modulate the expression level of collagen I (Fig. 2B).

### Pten is identified as a target gene of miR‐19b in cardiac fibroblasts

**Figure 3:**
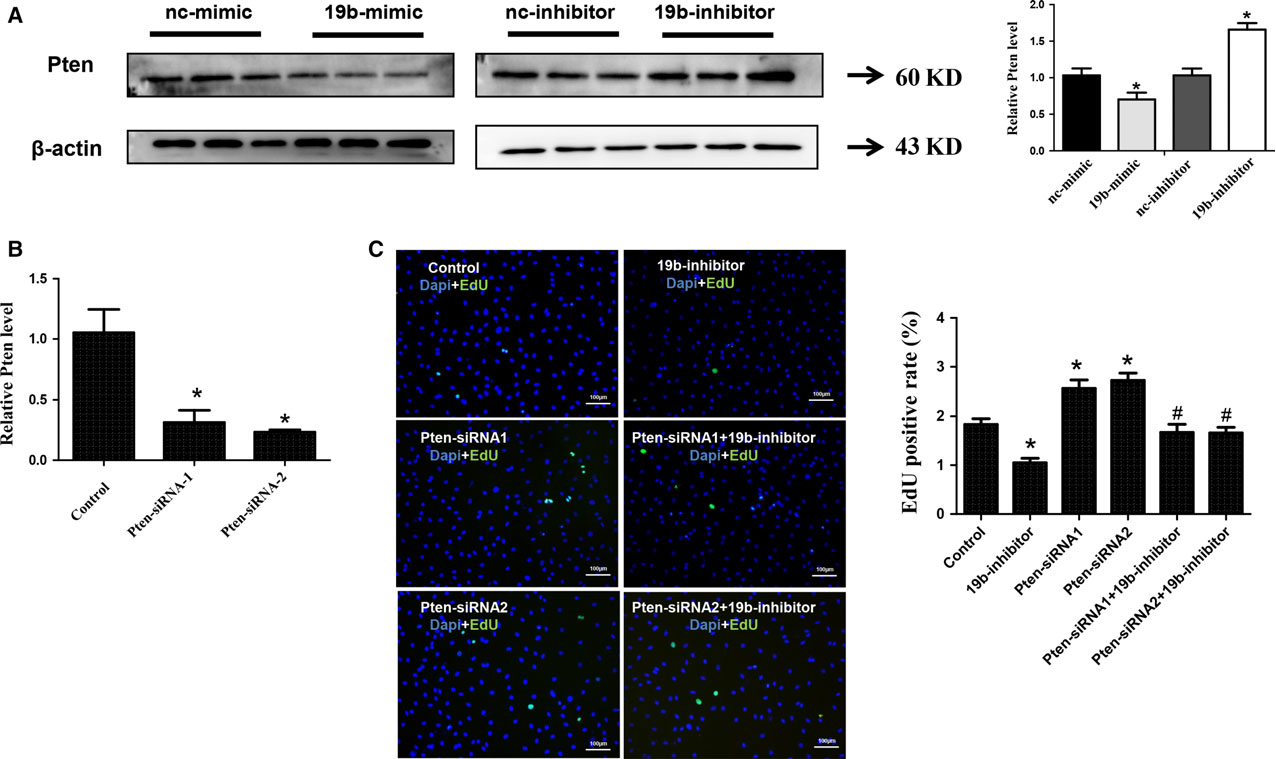
Pten is a target gene of miR‐19b involved in proliferation. (**A**) miR‐19b negatively regulated Pten at the protein level in cardiac fibroblasts. (**B**) Pten siRNAs decreased Pten at least at mRNA level. (**C**) Pten siRNAs significantly abolished the inhibitory effect of miR‐19b in proliferation of cardiac fibroblasts. Scale bar, 100 μm. *P < 0.05 *versus* control; #*P* < 0.05 *versus* miR‐19b inhibitor. Pten, Phosphatase and tensin homologue.

**Figure 4:**
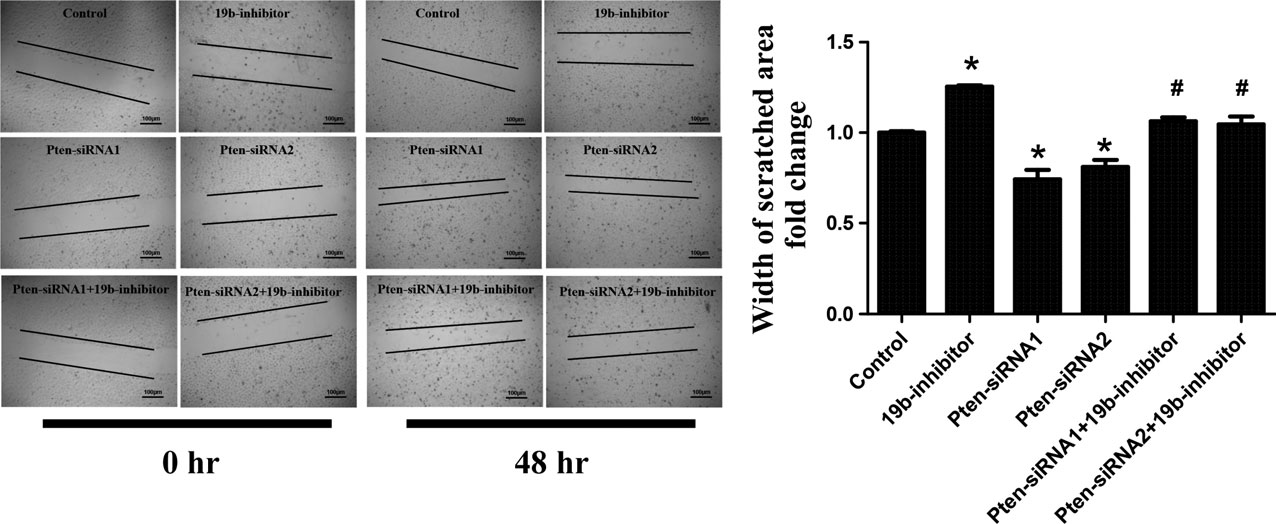
Pten is a target gene of miR‐19b involved in migration. Pten siRNAs significantly abolished the inhibitory effect of miR‐19b in migration of cardiac fibroblasts. Scale bar, 100 μm. *P < 0.05 *versus* control; #*P* < 0.05 *versus* miR‐19b inhibitor. Pten, Phosphatase and tensin homologue.

Phosphatase and tensin homologue (Pten) is a well‐known target gene of miR‐19b and has recently been found to be a target gene of miR‐19b in H9C2 cardiomyocytes 27. However, due to the cell‐specific effects of miRNA 13, 20, 21, 31, if Pten is a target gene of miR‐19b in cardiac fibroblasts is unclear. To investigate the relationship between miR‐19b and Pten in cardiac fibroblasts, we transfected cardiac fibroblasts with miR‐19b mimics and inhibitors and determined the protein expression level of Pten using Western blotting. We found that miR‐19b mimics down‐regulated while miR‐19b inhibitors up‐regulated Pten at the protein level (Fig. 3A). Two siRNAs for Pten were used in this study to exclude the off‐target effects. Both Pten siRNAs used here could decrease Pten at least at mRNA level (Fig. 3B). Thus, in the following studies, Pten siRNAs were used to further investigate if Pten was responsible for the effect of miR‐19b in cardiac fibroblast proliferation and migration. As determined by EdU staining, silencing Pten was able to increase cardiac fibroblasts proliferation and migration, while the inhibitory effect of miR‐19b inhibitor in both cardiac fibroblast proliferation and migration could be blocked by Pten siRNAs (Fig. 3C and Fig. 4). These results indicate that Pten is a target gene of miR‐19b responsible for the effects of miR‐19b in cardiac fibroblasts.

## Discussion

Cardiac fibrosis is the final most common pathway of lots of cardiac disorders and diseases 21. Unfortunately, currently no specific treatment has been approved for cardiac fibrosis worldwide 21. Thus, new therapeutics for cardiac fibrosis is highly desirable 20. Here, we found that miR‐19b was able to promote cardiac fibroblast proliferation and migration, and Pten was identified as a target gene of miR‐19b responsible for the effects of miR‐19b in cardiac fibroblasts. The miR‐19/Pten axis might be a novel therapeutic target for cardiac fibrosis.

It should be noted that although we consider reactive fibrosis to be undesirable within viable myocardium, fibrosis sometimes is desirable 30. For example, replacement fibrosis has been considered as an essential initial step for heart repair, and it is also important for compensating the limited regenerative capacity of the adult heart in damage 30. As miR‐19b could improve cell survival and protect H_2_O_2_‐induced apoptosis in H9C2 cardiomyocytes 27, it is highly interesting and urgent to detect the effects of miR‐19b in cardiac remodelling and heart failure *in vivo*.

Emergency evidence have proved that abnormal expressions of miRNAs were linked to cardiac fibrosis, including miR‐1, miR‐21, miR‐22, miR‐24, miR‐26a, miR‐101, miR‐652, *etc*. 19, 21, 32. In this study, we found that miR‐19b could promote cardiac fibroblast proliferation and migration, while miR‐19 has also been proved to be able to protect cardiomyocyte apoptosis and improve cell survival 27. This is not a unique case as miRNA exerts biological effects in a cell‐specific manner 27. For example, miR‐21 inhibition has been reported to be able to reduce cardiac fibrosis, while it (miR‐21 inhibition) may also enhance cardiomyocyte hypertrophy 5, 6, 19, 21, 32. Besides that, similar phenomenon has been observed with miR‐24 and miR‐15 19. Interestingly, miR‐19b has been reported to blunt the activated hepatic stellate cell phenotype by morphological assessment and decreased α‐SMS expression by targeting TGFβRII 33. Moreover, miR‐19b has been found to reduce hepatic stellate cell proliferation by targeting GRB2 in hepatic fibrosis models both *in vivo* and *in vitro* 34. This indicates a tissue‐specific role of miR‐19b. Thus, considering the fact that off‐target effects or opposing effects of miRNAs exist in different cells and tissues, *in vivo* studies are needed to test if enhancing or inhibiting a miRNA could be viewed as a useful therapy for cardiac fibrosis treatment. Also, it is extremely important that novel cardiovascular therapy delivery systems by targeting a specific cell population are supposed be developed to optimize miRNA therapy strategies in cardiac repair.

Several limitations of this study should be highlighted. First, this study investigated the effects of miR‐19b in cardiac fibroblasts in baseline condition. It would be interesting to explore the effects of miR‐19b in cardiac fibroblasts under stress such as TGF‐β1 and angiotensin II stimulation. Second, the upstream molecules responsible for the inducible effects of miR‐19b in cardiac fibroblasts are unclear. Third, though beyond the scope of this study, it is highly urgent and important to investigate if miR‐19b inhibition *in vivo* is beneficial or harmful in cardiac remodelling and heart failure in the future.

In conclusion, this study indicates that miR‐19b promotes cardiac fibroblast proliferation and migration by targeting Pten. The effects of pharmacological inhibition of miR‐19b in cardiac remodelling and heart failure *in vivo* need to be determined in the future.

## Conflict of interest

The authors declare that there are no conflicts of interest.
